# Spouses’ Emotion Regulation Skills and Perceived Partner Emotional Support in the Context of Early Stage Dementia

**DOI:** 10.1093/geroni/igaa057.3339

**Published:** 2020-12-16

**Authors:** Amanda Piechota, Joan Monin

**Affiliations:** 1 Yale School of Public Health, New Haven, Connecticut, United States; 2 Yale University, New Haven, Connecticut, United States

## Abstract

Within older adult marriages, spouses often help regulate one another’s emotions, especially in times of stress (Monin, 2016). Although research shows that better emotion regulation skills are associated with better social skills (Lopes, 2005), less is known about whether better emotion regulation skills in one spouse are associated with greater perceived relationship quality from their partner’s point of view. Further, very little is known about these dynamics in the context of early stage dementia in which both spouses may be feeling more stress over time and need support from one another. In the present study, we hypothesized that (a) one spouse’s greater emotion regulation skills would be associated with their own greater perceived emotional support from their partner and (b) their partner would also report greater perceived emotional support. We made the same hypothesis for both the person with dementia and their spouse without dementia. We used self-report baseline data from an intervention study of 45 older adult married couples (N=90) where one spouse has dementia. Spouses completed questionnaires that measured their emotional regulation habits (DERS; Gratz & Roemer, 2004) and perceived emotional support from their partner (Feeney & Collins, 2000). Results from the actor partner interdependence model showed that when both partners had high emotional regulation skills there were the highest levels of perceived partner support for each dyad member (B=-.02, SE=.01, t(38.83)=-2.37, p=.023). Findings suggest couple-focused interventions to enhance emotion regulation skills are important for maintaining relationship quality in the early stages of dementia.

